# Supplementary material for: Soil Pollution Characteristics and Microbial Responses in a Vertical Profile with Long-Term Tannery Sludge Contamination in Hebei, China
Source: Int J Environ Res Public Health. 2019 Feb 15;16(4):563. doi: 10.3390/ijerph16040563 (PMC6407015; doi:10.3390/ijerph16040563)
Supplement: Supplementary file 1 [file ijerph-16-00563-s001.pdf]

Supplementary Materials: The following are available online at www.mdpi.com/xxx/s1, Figure S1: Fourier transform infrared spectroscopy of the tannery sludge leachate., Table S1: Particle classification of soils according to the standard of the International Society of Soil Science (ISSS).

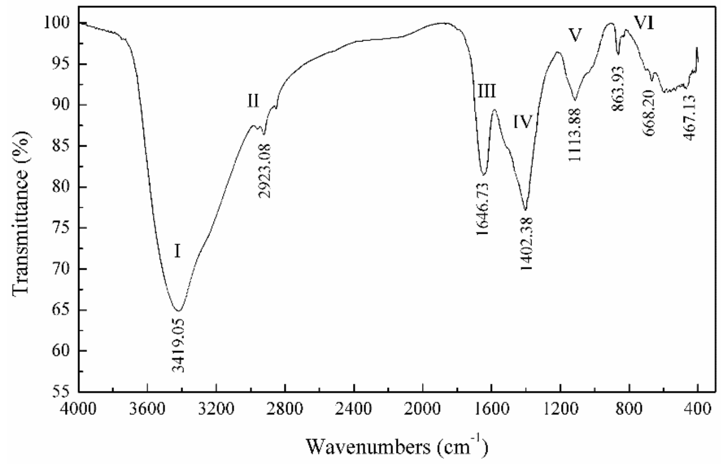

Figure S1. Fourier transform infrared spectroscopy of the tannery sludge leachate.

I, scanned area: 3600–3200 cm^{-1}, the stretching vibration band of O-H and the N-H groups in complexes of carboxylic acid, phenols, and the organic amine; II, scanned area: 3000–2800 cm^{22121}, the stretching vibration band of $-CH_3$,$-CH_2-$ and -C-H- groups in the aliphatic compounds; III, scanned area: 1700–1635 cm^{-1}, the stretching vibration band of -COOH-, O-H and -C=O groups in complexes of carboxylic acids, amides and ketones; IV, 1450–1360 cm^{-1}, the stretching vibration band of $-CH_3$ and $-CH_2-$ in the aliphatics; V 1070–1150 cm^{-1}, the stretching vibration band of C-O-C in the aliphatics; VI scanned area:870–640 cm^{-1}, the stretching vibration band of C-H groups.

Table S1. Particle classification of soils according to the standard of the International Society of Soil Science (ISSS).

Samples	Depth/cm	<0.002 mm/%	0.002~0.02 mm/%	0.02~2 mm/%
TS	surface	4.83	37.83	57.34
S1	0~20	1.17	13.76	85.07
S2	20~40	4.76	44.32	50.92
S3	40~60	4.61	36.99	58.40
S4	60~80	4.27	42.38	53.35
S5	80~100	3.49	31.61	64.90
S6	120~150	8.28	48.95	42.77
S7	150~180	2.43	37.28	60.29
S8	180~200	11.64	39.37	48.99
US1	0~20	5.01	46.48	48.51
